# Comparison of various predictive tools in predicting risk of cerebrospinal fluid diversion post-resection of posterior fossa tumors: A systematic review

**DOI:** 10.12669/pjms.41.13(PINS-NNOS).13459

**Published:** 2025-12

**Authors:** Sundas Irshad, Amna Sohail, Raahim Bashir, Fiza Ismail, Sabeel Mahmood, Syeda Aleena Hassan, Sameed Safdar, Hania Fatima, Haseeb Mehmood Qadri, Sabino Luzzi, Asif Bashir

**Affiliations:** 1 Sundas Irshad, MBBS. Punjab Institute of Neurosciences, Lahore, Pakistan; 2 Amna Sohail, MBBS. Chengde Medical University, China; 3 Raahim Bashir, MBBS. CMH Medical and Dental College, Lahore, Pakistan; 4 Fiza Ismail, MBBS. Continental Medical College, Lahore, Pakistan; 5 Sabeel Mahmood, MBBS. Allama Iqbal Medical College, Lahore, Pakistan; 6 Syeda Aleena Hassan, MBBS. Fatima Jinnah Medical University, Lahore, Pakistan; 7 Sameed Safdar, MBBS. Allama Iqbal Medical College, Lahore, Pakistan; 8 Hania Fatima, MBBS. Aga Khan University, Karachi, Pakistan; 9 Haseeb Mehmood Qadri, MBBS. Punjab Institute of Neurosciences, Lahore, Pakistan; 10 Sabino Luzzi, MBBS, MD, FACS, PhD. University of Sassari, Sardinia, Italy; 11 Asif Bashir, MBBS, MD, FACS, FAANS (Diplomate American Board of Neurosurgery). Punjab Institute of Neurosciences, Lahore, Pakistan

**Keywords:** Artificial intelligence, Hydrocephalus, Humans, Infratentorial neoplasm

## Abstract

**Background & Objective:**

Posterior fossa tumors (PFTs) frequently cause hydrocephalus (HCP), requiring permanent cerebrospinal fluid (CSF) diversion post resection in both pediatric and adult patients. We aimed to compare the performance of various tools in predicting the risk of postoperative hydrocephalus and improving prediction for better neurosurgical decision-making.

**Methodology:**

A comprehensive literature search was conducted across Google Scholar and PubMed database adhering to Preferred Reporting Items for Systematic Review and Meta-analyses (PRISMA) guidelines using keywords such as posterior fossa tumors, hydrocephalus, CSF diversion, and predictive models. A total of ten original articles with a sample size of 1597 from 2021 to 2025 were selected for data extraction. Study quality evaluation was executed via PROBAST tool.

**Results:**

The cumulative mean ages were 7.18±1.64 years for pediatric patients and 53.07±0.81 years for adults. Pediatric patients accounted for 67.31% (1075) patients while adults accounted for 19.41% (402) patients. Preoperative hydrocephalus was present in 52.4% (833) patients, out of which 22.9% (367) required CSF diversion post resection. Pooled post-operative shunt rates revealed higher shunt rate 53.9% (715) in patients with preoperative hydrocephalus than those without 13% (80). Logistic regression was used in 88.8% (8) of the identified models while AI based model demonstrated best performance (AUC = 0.938).

**Conclusion:**

Of all the predictive models developed, till now, to predict the need for CSF diversion after PFT resection, artificial intelligence-based model shows superior accuracy for improving hydrocephalus risk prognostication. Apart from clinical, demographic, surgery-related and radiological predictors incorporated in conventional predictive models, the artificial-intelligence based model improves risk prediction by utilizing complex patterns in intraoperative and postoperative imaging of patients.

## INTRODUCTION

Posterior fossa tumors (PFTs) can lead to obstructive or communicating hydrocephalus (HCP) due to their proximity to the cerebral aqueduct and fourth ventricle. Surgical resection is the primary treatment for PFTs and typically resolves hydrocephalus in most cases.^1^ However, persistent or recurrent HCP following resection remains a significant challenge, affecting 1.2–6.9% of adult patients and about 30% of pediatric patients.^1^ In such cases, permanent cerebrospinal fluid (CSF) diversion through shunt, external ventricular drain (EVD) or endoscopic third ventriculostomy (ETV) becomes necessary.^2^ Identifying patients at risk for permanent CSF diversion remains a critical challenge, as untreated hydrocephalus can lead to severe neurological complications, prolonged hospital stays, and increased morbidity in 10-30% of the patients.^3^

Despite advancements, the literature on CSF diversion prediction remains limited, with most studies focusing on factors such as tumor location, preoperative hydrocephalus, and extent of resection. Additional predictors of post-resection hydrocephalus include younger age at presentation, severity and duration of hydrocephalus, ventricular volume, tumor histology, presence of metastasis, use of external ventricular drainage, and postoperative complications like infection, pseudo meningocele, or intraventricular hemorrhage on imaging.^4^ Due to the limited understanding of the underlying mechanisms contributing to disrupted cerebrospinal fluid (CSF) circulation despite tumor resection and the removal of the primary obstructive element, the optimal approach to managing hydrocephalus remains a subject of ongoing debate.^5^ Therefore, a deeper understanding of these risk factors, along with the development of reliable risk stratification tools, is essential to better guide clinical decision-making.^6^,^7^

To address this complexity, we have compared the performance of various predictive models developed via statistical methods and machine learning to calculate the risk of CSF shunting post operatively. We have also compared the performance of different variables to assess their contribution to the development of this complication. According to our knowledge, this is the first study conducted across PubMed and Google Scholar that has evaluated the performance of all existing models and variables, providing a comprehensive review of the predictive performance of each available tool to enhance neurosurgical decision-making and ideally promote better outcomes.

## METHODOLOGY

A systematic review was conducted on the topic “Comparison of Various Predictive Tools in Predicting Risk of Cerebrospinal Fluid Diversion Post-Resection of Posterior Fossa Tumor” as per guidelines of Preferred Reporting Items for Systematic reviews and Meta-Analysis (PRISMA).^8^ Our systematic review was registered with PROSPERO (CRD420251019237). PubMed and Google Scholar database was used to search the included articles from 2021-2025. Quality assessment of articles was performed. The Boolean strategy employed terms such as “AND” and “OR” with the following combination of keywords, “artificial intelligence” OR “machine learning” OR “deep learning” OR “AI” OR “ML”, AND “CSF diversion” OR “hydrocephalus” OR “shunt placement” AND “posterior fossa tumor” OR “cerebellar tumor” AND “prediction”.

### Inclusion criteria:

- Original articles
- English Language
- Human beings
- Data with complete information.

### Exclusion criteria:

- Letters to the editors
- Editorials
- Animal and cadaveric studies
- Non-English studies

**Fig.1:**
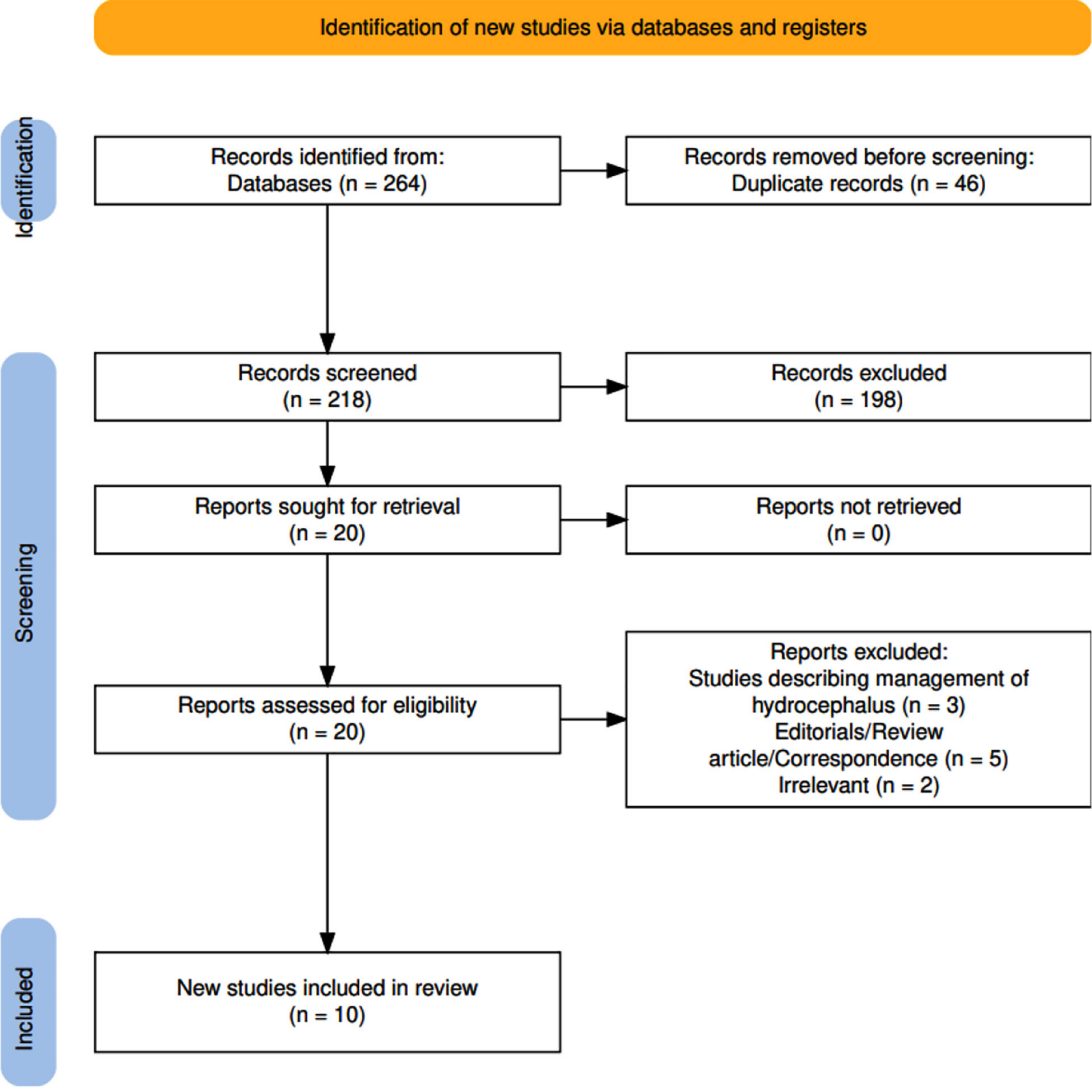
Preferred Reporting Items for Systematic Review and Meta-analyses (PRISMA) flowchart for search strategy and the quality assessment of included studies.

A total of 264 articles were retrieved, after screening and removal of 46 duplicates, 10 studies from 2021 to 2025 were included. The PRISMA flowchart is illustrated below (Fig.1).

Literature search was conducted by two independent reviewers (RB and SAH). FI and SI verified the data to minimize bias. Quality of the articles was evaluated by HF and verified by AS using PROBAST (Prediction model Risk of Bias Assessment Tool).^9^ The PROBAST checklist is mentioned in Appendices. The data regarding study characteristics, patient demographics, clinical features, model characteristics (components, performance metrics) and types of predictor variables was extracted in Microsoft Excel.

### Data analysis:

Descriptive statistics in the form of measure of central tendency, frequencies and percentages were calculated by using Microsoft Excel. A summary of the literature included in this systematic review is given in (Table-I).

**Table-I:** Details of included studies.

Study by	Study Title	Country of Study	Year of Publication	Study design	Aim of Study
Bray et al.^10^	Artificial neural networks predict the need for permanent cerebrospinal fluid diversion following posterior fossa tumor resection.	USA	2022	Retrospective cohort, Multi-center study	To develop an algorithm using AI that can reliably predict the need for VPS after PFT surgery.
Gyasi et al.^11^	Validating the modified Canadian Preoperative Prediction Rule for Hydrocephalus for accurate hydrocephalus prediction in a state-wide paediatric brain tumor cohort.	USA	2025	Retrospective cohort Single-institution study	To externally validate the mCPPRH in a cohort of 113 pediatric patients with PFTs.
Pitsika et al.^12^	A validation study of the modified Canadian Preoperative Prediction Rule for Hydrocephalus in children with posterior fossa tumors.	UK	2021	Retrospective cohort Single-institution study	To externally validate mCPPRH.
Park et al.^13^	Ventriculomegaly and postoperative lateral/third ventricular blood as predictors of cerebrospinal fluid diversion following posterior fossa tumor resection.	USA	2021	Retrospective cohort analysis, Single-institution study	To determine the clinical and radiographic predictors for CSF diversion in children following posterior fossa tumor resection.
Zhang et al.^14^	Contribution of tumor characteristics and surgery-related factors to symptomatic hydrocephalus after posterior fossa tumor resection: a single-institution experience.	China	2022	Retrospective Cohort, Single-institution study	To analyse the influence of tumor characteristics and surgery-related factors on postoperative hydrocephalus in Chinese children.
Ying et al.^15^	Risk factors for postoperative ventriculoperitoneal shunt requirement in pediatric patients with brain tumors invading or adjacent to CSF circulation pathways.	China	2024	Retrospective Cohort, Single-institution study	To explore perioperative risk factors associated with postoperative ventriculoperitoneal shunt placement for tumors located at or adjacent to the CSF circulation pathway.
Bernstein et al.^16^	Characterizing the association between CSF biomarkers and risk for ventriculoperitoneal shunt following posterior fossa tumor resection in a case-control study.	United States	2024	Retrospective cohort, Multi-center study	To evaluate which CSF laboratory values are associated with permanent CSF diversion following PFT resection in adults.
Zhou et al.^17^	A nomogram for predicting post-operative hydrocephalus in children with medulloblastoma.	China	2023	Retrospective cohort Single-institution study	To establish a novel model for predicting the development of post-operative hydrocephalus in children with medulloblastoma.
Darshan et al.^18^	Analysis of evolution of hydrocephalus in posterior fossa tumors and validation study of the modified Canadian preoperative prediction rule for hydrocephalus in children and Frankfurt grading system for prediction of cerebrospinal fluid diversion in adults with posterior fossa tumors.	India	2023	Retrospective cohort, Single-institution study	To analyze the evolution of hydrocephalus and enumerate its predictive factors in posterior fossa tumors in children and adults along with validating the mCPPRH and Frankfurt grading systems as tools to predict CSF diversion in children and adults, respectively.
Hu et al.^19^	A nomogram for predicting post-operative hydrocephalus in children with medulloblastoma.	China	2023	Retrospective cohort, Single-institution study	To identify the risk factors for postoperative hydrocephalus and the need for ventriculoperitoneal (VP) shunt after posterior fossa tumor (PFT) resection in pediatric patients and establish a predictive model.

***Abbreviations:*** mCPPRH: modified Canadian Preoperative Prediction Rule for Hydrocephalus.

## RESULTS

A total of 10 studies comprising 1597 participants were included in our review (2190 if training cohorts are included). Of these 1597 participants, there were 50.9% (813) males and 47.21% (754) females, excluding 1.8% (30) patients whose gender was unspecified. The cumulative mean age was 7.18±1.64 years in pediatric age group and 53.07±0.81 years in adults. Majority of the participants included in our review were from the pediatric age group (<18 years) i.e., 67.31% (1075) were children while 19.41% (402) were adults, while in the remaining participants, age group was unspecified. All the studies included in our review were retrospective, 100% (10), of which 20 % (2) were conducted at multiple centers whereas data was collected from single centers in 80% (8) of included studies. According to the regional distribution, most of the studies were conducted in developed countries like USA, UK and China i.e., 4 (40%), 1 (10%), 4 (40%) respectively whereas only 1(10%) was from India, a developing country.

Majority of the included studies aimed to identify new predictors and develop novel models for prediction of hydrocephalus after posterior fossa tumor resection i.e., 70% (7) studies whereas 10% (1) validated previously existed models and 20% (2) followed the combined approach of both development of new model along with validation of previously existent models.

According to the tumor type, most of the study population included in our review had Medulloblastoma, 26.61% (425), followed by Astrocytoma 13.9% (222), Schwannoma 6.9% (111), Ependymomas 5.57% (89), Hemangioblastoma 2.69% (43) and others while 17.09% (273) had metastasis at the time of resection except in Zhang et al.^14^ and Bray et al.^10^ where exact number of tumor types studied were not specified.

Preoperative hydrocephalus was present in 52.4% (838) patients while postoperative hydrocephalus requiring CSF diversion were reported in only 22.9% (367) patients. Of all the studies included in our review, the data for postoperative shunting categorized according to presence of preoperative hydrocephalus was reported in only six studies. Among those six studies, 53.9% (715) patients had preoperative hydrocephalus. The pooled shunt rates in patients suffering from preoperative hydrocephalus were 25.1% (180), whereas it was lower in patients without preoperative hydrocephalus i.e., 13% (80) patients (Table-II).

**Table-II:** Shunt requirements in hydrocephalus.

Study by	N	Preoperative hydrocephalus, % (n)	Postoperative hydrocephalus, % (n)	Percentage (%) of shunt requirement in patients with preoperative hydrocephalus	Percentage (%) of shunt requirement in patients without hydrocephalus
Bray et al.^10^	90	34.4 (31)	13.3 (12)	-	-
Gyasi et al. 11	113	77.8 (88)	30.9 (35)	37.5 (33)	8 (2)
Pitsika et al.^12^	75	73.3 (55)	10.6 (8)	9.09 (5)	15 (3)
Park et al.^13^	63	82.5 (52)	41.2 (26)	-	-
Zhang et al.^14^	197	26.3 (52)	15.2 (30)	17.3 (9)	14.4 (21)
Ying et al.^15^	265	49.8 (132)	14.3 (38)	16.7 (2)	27 (36)
Bernstein et al.^16^	89	29.2 (26)	70.7 (63)	-	-
Zhou et al.^17^	29	48.2 (14)	20.6 (6)	-	-
Darshan et al.^18^	459	48.1 (221)	26.1 (120)	46.6 (103)	7.1 (17)
Hu et al.^19^	217	76.9 (167)	13.3 (29)	16.7 (28)	2 (1)

**Fig.2:**
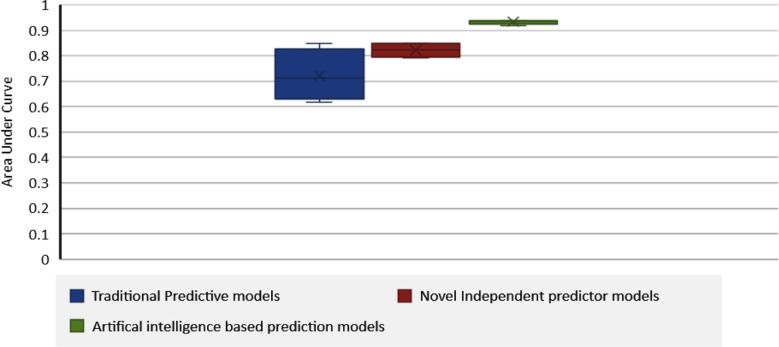
Area under curves of various predictive models.

A total of nine predictive models were identified from ten included studies including two traditional predictive models mCPPRH (modified Canadian Preoperative Prediction Rule for Hydrocephalus) grading system and Frankfurt grading system, five independent predictors-based models, one nomogram-based model and one artificial intelligence based predictive model. Majority of the models were based on logistic regression analysis 90% (8), whereas artificial intelligence technology was employed in only 10% (1) of all predictive models. The Area under curve (AUC) of predictive models ranged from 0.659 to 0.938. Out of these, the maximum value of AUC was reported for artificial intelligence-based model (AUC= 0.938), followed by 0.847 which was the maximum value of AUC reported for traditional logistic regression-based models and 0.849 for novel logistic regression-based models, and AUC values for two independent predictor-based models were missing (Fig.2).

The most frequently described model components were age, tumor location and occurrence of preoperative hydrocephalus, each present in 66.6% (6) of models, followed by postoperative intraventricular blood in 55.5% (5) models and extent of resection in 33.3% (3) models (Table-III).

**Table-III:** Predicted model characteristics in included studies.

Study by	Model Name	Model Type	Model Components	AUC
Bray et al.^10^	ANN	Artificial intelligence-based model	Patient demographics,	0.938
			Pre-operative and post-operative MRI,	
			Surgical resection,	
			Surgical complications	
Gyasi et al.^11^	Modified mCPPRH grading system	Logistic regression	Age <5 years, Moderate or severe hydrocephalus.	0.764
Pitsika et al.^12^	mCPPRH grading system	Logistic regression	Age < 2 years,	0.618
			Moderate/severe hydrocephalus, Trans-ependymal edema, Preoperative tumor diagnosis,	
			Presence of metastasis.	
Park et al.^13^	Novel-prediction model	Logistic regression	Frontal occipital horn ratio,	0.79
			MRI blood presence in ventricles	
Zhang et al.^14^	Novel-prediction model	Logistic regression	Gender,	0.804
			Tumor location,	
			Tumor metastasis,	
			Post-operative intraventricular blood volume,	
			Radiotherapy	
Ying et al.^15^	Novel-prediction model	Logistic regression	Medulloblastoma,	-
			Third or lateral ventricle, Post-operative lateral or third ventricle blood,	
			Post-operative subdural hygroma	
Bernstein et al.^16^	Novel-prediction model	Logistic regression	Non-clear CSF appearance, Postoperative CSF glucose	-
Zhou et al.^17^	Nomogram	Logistic regression	Evans index,	0.849
			Trans-ependymal volume,	
			Intra-ventricular blood volume, Superior invasion,	
			Caudal invasion,	
			Anterior invasion and	
			Extent of resection	
Darshan et al.^18^	mCPPRH grading system	Logistic regression	Age (<2 years),	0.659
			Moderate/severe hydrocephalus,	
			Pre-operative tumor diagnosis, Trans ependymal edema.	
Darshan et al.^18^	Frankfurt grading system	Logistic regression	Pre-operative hydrocephalus,	0.847
			Periventricular CSF capping,	
			Patient positioning,	
			Expected extent of resection for intraparenchymal tumors,	
			Pre -operative hydrocephalus,	
			Tumor location,	
			Perilesional edema for extra parenchymal tumors	
Hu et al.^19^	Novel-prediction model	Logistic regression	Intraoperative blood loss,	0.842
			Age<3 years,	
			Tumors at 4th ventricle	

The significant predictor variables identified in our included studies were demographics (age, gender), clinical variables (preoperative hydrocephalus, tumor metastasis), tumor-related predictors (tumor diagnosis, tumor location, tumor invasion), radiological variables (perilesional edema, periventricular capping, fronto-occipital horn ratio), surgery-related features (intraoperative blood loss, extent of surgical resection, postoperative intraventricular hematoma, postoperative CSF glucose, non-clear CSF appearance and subdural hygroma) (Table-IV).

**Table-IV:** Significant predictors of postoperative hydrocephalus after posterior fossa tumor resection

Variables	Study by	Odds Ratio (OR)	95% CI (Lower limit)	95% CI (Upper limit)	p-value
***Demographic & Baseline Clinical predictors***					
Age	Darshan et al.^18^	0.956	0.931	0.982	0.001
Age<3 years	Hu et al.^19^	3.76	1.294	10.927	0.015
Gender	Darshan et al.^18^	2.009	1.025	3.937	0.042
Preoperative Hydrocephalus	Darshan et al.^18^	0.038	0.014	0.105	0.001
	Darshan et al.^18^	0.042	0.012	0.148	0.001
	Gyasi et al.^11^	6.37	1.71	41.55	0.02
Tumor metastasis	Zhang et al.^14^	3.463	1.137	10.549	0.029
***Tumor-related predictors***					
Tumor diagnosis (Medulloblastoma)	Ying et al.^15^	4.15	1.74	9.91	0.001
	Darshan et al.^18^	0.082	0.019	0.348	0.001
Tumor Location					
Fourth ventricle	Hu et al.^19^	7.697	2.595	22.835	0.001
Lateral/3rd ventricle tumor	Ying et al.^15^	4.07	1.33	12.3	0.014
Tumor Invasion					
Superior invasion	Zhou et al.^17^	7.467	1.738	32.091	0.007
Caudal invasion	Zhou et al.^17^	4.56	2.205	21.238	0.025
***Radiological predictors***					
FOHR	Park et al.^13^	2.9	1.3	6.8	0.02
Periventricular capping in children (Darshan et al.)	Darshan et al.^18^	0.118	0.049	0.284	0.001
***Surgery-related predictors***					
Extent of resection in Extra-axial tumors	Darshan et al.^18^	0.082	0.031	0.224	0.001
Extent of resection in Intra-axial tumors	Darshan et al.^18^	0.028	0.007	0.115	0.001
Intraoperative Blood loss	Hu et al.^19^	1.601	1.196	2.143	0.002
Intraventricular blood volume/hematoma	Zhou et al.^17^	4.536	1.032	19.937	0.045
	Ying et al.^15^	3.36	1.53	7.38	0.003
	Zhang et al.^14^	4.212	1.595	11.122	0.004
	Park et al.^13^	20.2	2.9	423.1	0.01
CSF appearance (non-clear)	Bernstein et al.^16^	4.15	1.47	12.56	0.009
postoperative CSF glucose	Bernstein et al.^16^	1.03	1.01	1.07	0.031
Postoperative Subdural hygroma	Ying et al.^15^	2.37	1.1	5.12	0.024

The analysis of predictor variables for development of hydrocephalus after posterior fossa tumor resection showed that younger age, female gender, presence of high grade tumor as tumor diagnosis such as Medulloblastoma, extension of tumor onto ventricles (third, fourth or lateral ventricles), superior and caudal invasion of tumor, increased FOHR, postoperative intraventricular blood , non-clear CSF appearance, CSF glucose , postoperative subdural hygroma and occurrence of tumor metastasis are all associated with significantly increased odds of developing hydrocephalus postoperatively i.e., OR >1, p<0.05. Postoperative intra-ventricular blood volume showed the highest odds for development of hydrocephalus or need for CSF diversion after posterior fossa tumor resection with the odds ratio (OR) ranging from 3.36 (95% CI: 1.53-7.38, p=0.003) to 20.2 (95% CI: 2.9-423.1, p=0.01).

The posterior fossa tumors extending onto fourth ventricle or located in fourth ventricles had a higher odd of developing postoperative hydrocephalus in comparison to posterior fossa tumors located in lateral or third ventricles i.e., OR: 7.69, 95% CI: 2.59–22.83, p = 0.001 and OR: 4.07, 95% CI: 1.33–12.31, p = 0.014. Similarly, superior invasion of posterior fossa tumors had higher odds of developing postoperative hydrocephalus in comparison to caudal invasion i.e., OR: 7.46, 95% CI: 1.73–32.09, p = 0.007 and OR: 4.56, 95% CI: 2.20–21.23, p = 0.025.

Among the radiological predictors, higher FOHR showed a significantly higher odds of developing hydrocephalus postoperatively in comparison to periventricular capping in children which was associated with decreased likelihood of developing hydrocephalus postoperatively i.e., OR: 2.9, 95% CI: 1.3–6.8, p = 0.02 and OR: 0.11, 95% CI: 0.04-o.28, p = 0.001 respectively (Table-IV).

In surgery-related predictors, it was found that the greater the extent of resection, the lower was the likelihood of developing hydrocephalus postoperatively, in both intra-axial (OR: 0.028, 95% CI: 0.007–0.115, p = 0.001) and extra-axial tumors (OR: 0.082, 95% CI: 0.03-0.224, p = 0.001), although the predictive association was stronger in case of intra-axial tumors (Table-IV).

## DISCUSSION

This review aimed to identify the factors and various tools that may predict occurrence of hydrocephalus after posterior fossa tumor resection. The findings of our systematic review showed a slight male predilection at 50.9%. This is in contrast to a study on postoperative hydrocephalus after PFT surgery^5^ ,while another study shows an equal incidence of CSF diversion rates between males and females.^2^ Interestingly, Kumar et al.^20^ showed that almost twice as many men as women required CSF diversion within 30 days after resection.^20^ None of these studies demonstrated any statistical significance between gender and risk of CSF diversion after PFT resection, which, along with the heterogeneity in gender preponderance in the literature, suggests that gender does not affect the need for CSF diversion. On the whole, the majority of patients (67.3%) were children with a mean age of 7.18 years, while 19.4% were adults with a mean age of 53.07 years. This reflects the age distribution of PFT and hydrocephalus, both of which are well-known to disproportionately affect the pediatric population.

**Table-I:** APPENDICES PROBAST assessment of included studies.

Author	Risk of Bias according to PROBAST Domain				
	Participants	Predictors	Outcomes	Analysis	Overall
Bray et al.^10^	Low	Low	Low	Low	Low
Gyasi et al.^11^	Low	Low	Low	Unclear	Unclear
Pitsika et al.^12^	Low	Low	Low	High	High
Park et al.^13^	Low	Low	Low	Low	Low
Zhang et al.^14^	Low	Low	Low	Low	Low
Ying et al.^15^	Low	Low	Low	High	High
Bernstein et al.^16^	Low	Unclear	Low	Unclear	Unclear
Zhou et al.^17^	Low	Low	Low	High	High
Darshan et al.^18^	Low	Low	Low	High	High
Hu et al.^19^	Low	Low	Low	High	High

Central nervous system tumors are the most common pediatric solid malignancies, with PFT such as medulloblastoma, pilocytic astrocytoma, and ependymomas the most frequent neoplasms. 4,20 In the pediatric population, the frequency of hydrocephalus before PFT resection can range from 70-90%,^1^,^2^ compared to far lower rates of 10-21.4% in adults.^1^ Similarly, the incidence of postoperative hydrocephalus is lower in adults than in children,^1^-^3^ with hydrocephalus resolving after tumor resection in the vast majority of adults.^5^ Regarding specific tumor types, medulloblastoma (26.6%) was the most common tumor associated with CSF diversion in our study. This was followed by astrocytoma (13.9%) and schwannoma (6.9%). Indeed, medulloblastoma has been demonstrated to be a risk factor for both obstructive and communicating hydrocephalus^11^ as well as post-resection CSF diversion,^13^,^14^ and confers a point on the CPPRH.^11^,^12^

Hydrocephalus before and after PFT surgery holds an intricate relationship, both in its progression, and also with the need for postoperative CSF diversion. The average pooled preoperative hydrocephalus prevalence was 52.4% across all reviewed studies, decreasing to 22.9% postoperatively. This 56.3% change can most logically be attributed to removal of the source of ventricular obstruction (i.e., tumor). Similar to our results, Kombogiorgas et al.^21^ studied the predictive value of preoperative ventricular volume on the requirement for permanent CSF diversion after PFT and showed a 31% decrease in ventricular volume postoperatively compared to before resection. Moreover, their study showed that higher preoperative and postoperative ventricular volumes held a statistically significant predictive value for the need for permanent shunt placement.^21^,^22^ In our review, the pooled postoperative shunt rates of patients without preoperative hydrocephalus were almost half those of patients suffering from preoperative hydrocephalus (13% vs. 25.1%). This suggests that preoperative hydrocephalus may be a major predictor on the need for permanent CSF diversion after PFT surgery. This is corroborated by Gopalakrishnan et al.^23^ who noted that severe hydrocephalus at presentation was a risk factor for post-resection hydrocephalus. Indeed, both CPPRH and Frankfurt Grading Scale utilize preoperative hydrocephalus as a major criterion to identify patients at high-risk of post-resection hydrocephalus.^1^ However, it is important to consider that simply removing the nidus of ventricular obstruction may not be enough to restore normal CSF flow dynamics, as PFT resection may actually convert an obstructive hydrocephalus to communicating thereby still necessitating permanent CSF diversion.^11^ A multifaceted approach on a case-by-case basis should be employed when evaluating the possible need for permanent CSF diversion in patients with PFT and hydrocephalus.

Various predictive scores and statistical models were used to determine the risk of post-resection CSF diversion. These included traditional predictive models such as the CPPRH/modified CPPRH (mCPPRH) and the Frankfurt grading score, machine learning based artificial neural networks (ANN) models, and various novel prediction models incorporating multivariate logistic regressions and nomogram models. The artificial intelligence based model led the pack with an AUC of 0.938 as well as a 98.8% negative predictive value. This underscores the potential of ANN-based models to accurately predict the need for long-term CSF shunting after PFT surgery, with future advancements likely to enhance their diagnostic utility even further. Similar ANN have recently been shown to be effective tools in identifying patients at high-risk of CSF shunt failure.^3^ Authors of this study believe that the need for advanced infrastructure, technical expertise and high-quality images required in AI-based prediction models might limit its real-word applicability in resource limited settings, a higher predictive accuracy in this review necessitates the need for diverting resources in similar studies to improve patient outcomes.

Across all studies, common model components (i.e. variables) included frontal occipital horn ratio (FOHR), tumor type and location, presence of preoperative hydrocephalus, presence of trans ependymal edema, and degree of intraventricular hemorrhage (IVH). As mCPPRH and a novel prediction model by Hu et al.^19^ incorporated age as a predictor variable, the accuracy of these models in adult population might be limited.

Although, preoperative hydrocephalus was significantly associated with the risk of postoperative hydrocephalus requiring permanent CSF diversion. the direction of association was towards causality (positive) in some studies and protective (negative) in others. In this regard, the Frontal and Occipital Horn Ratio (FOHR) may serve as a more meaningful marker of preoperative hydrocephalus, offering a straightforward radiological measure with reduced interrater variability compared to a clinical diagnosis of hydrocephalus. Park et al.^13^ noted the FOHR to significantly increase the odds of postoperative hydrocephalus (OR=2.9, p=0.02). Previous work^22^-^24^ has attested to the FOHR having the best correlation with ventricular size, and it has the additional benefit of being independent of age.^22^ Additionally, tumors invading the fourth ventricle and lateral/third ventricles had OR of 7.697 and 4.07 at p=0.001 and p=0.014, respectively. This suggests that midline tumors abutting into the ventricular system have a significantly greater likelihood of causing post-resection hydrocephalus necessitating CSF diversion. This aligns with the findings of Zhang et al.^5^, who identified ventricular system infiltration by tumor as an independent risk factor for postoperative CSF shunt placement (OR = 58.5, P < 0.001), and Anetsberger et al.,^4^ who concluded that midline tumor location was associated with an increased risk of perioperative complications, including persistent hydrocephalus. Our review showed that postoperative intraventricular hemorrhage consistently displayed the greatest odds of post-resection hydrocephalus and permanent CSF shunting, with the OR ranging from 3.36 (95% CI: 1.53-7.38, p=0.003) to 20.2 (95% CI: 2.9-423.1, p=0.01). This is similar to other work where the presence of postoperative IVH was a significant predictor of postoperative shunting.^5^ Trans ependymal edema, while not included in our review, is another consistently demonstrated predictor, with Kumar et al.^20^ concluding periventricular lucency on preoperative imaging to be the strongest independent predictor of CSF diversion in pediatric PFT within one month of tumor excision.

### Limitations:

A few limitations of this review should be mentioned. Only one article on AI-based prediction models could be included in this review for comparison with statistical-based models due to the paucity of existing research on the subject. Due to the heterogeneity of the data, a meta-analysis could not be conducted. Five of the ten included studies had high risk of bias, and two were unclear, mostly due to lack of internal validation. However, these were included due to limited literature availability. Most of the included studies were from developed countries which might have introduced geographical bias. Additionally, literature search was limited to open-access databases only, hence the risk of publication bias should be considered.

## CONCLUSION

Although conventional predictive models like mCPPRH and Frankfurt grading system have laid foundational groundwork for prediction of hydrocephalus after posterior fossa tumor resection, limited integration of intraoperative and postoperative imaging features underscores the superior performance of ANN-based models suggesting a promising avenue for future research and clinical applications. A reliable, standardized prediction model–ideally one that incorporates demographic, clinical, radiological, and intraoperative features–may enable timely risk stratification, reduce unnecessary interventions, and guide more personalized management strategies for hydrocephalus following PFT resection.

### Clinical recommendations:

We suggest incorporating a multidisciplinary and multifaceted approach when managing hydrocephalus in PFT patients. Demographic and baseline clinical features, radiological parameters, and surgical factors should all be assessed when predicting the need for permanent CSF diversion after PFT surgery. The variables of greatest predictive strength include the FOHR, midline tumor location and/or ventricular system invasion, intraventricular hemorrhage, and younger age. Traditional predictive models such as the CPPRH/ mCPPRH in children and the Frankfurt grading score in adults should be utilized when possible due to their generally satisfactory level of validation and ease of use. However, avant-garde predictive tools–including novel nomograms and particularly machine learning based ANN models–should be the focus of future clinical research and validation efforts to facilitate their integration into contemporary clinical practice.

### Author`s Contribution:

**SI:** Concept of the study and critical review of the manuscript.

**AS:** Data interpretation and drafted the manuscript.

**RB, SM, SAH and SS:** Data acquisition, and drafted the manuscript.

**FI:** Data interpretation and critically reviewed the manuscript.

**HF:** Data acquisition, critical review and drafted the manuscript.

**HMQ, SL and AB:** Supervision, Design of the study, and critically reviewed the manuscript.

All the authors have read and approved the final manuscript and are responsible and accountable for the accuracy and integrity of the work.
